# Recent Patents in Allergy/Immunology: Use of arginase inhibitors in the treatment of asthma and allergic rhinitis

**DOI:** 10.1111/all.13770

**Published:** 2019-04-10

**Authors:** Herman Meurs, Johan Zaagsma, Harm Maarsingh, Marcel van Duin

**Affiliations:** ^1^ Department of Molecular Pharmacology Groningen Research Institute for Asthma and COPD University of Groningen Groningen The Netherlands; ^2^ Department of Pharmaceutical Sciences Lloyd L. Gregory School of Pharmacy Palm Beach Atlantic University West Palm Beach Florida; ^3^ Ferring Pharmaceuticals San Diego California

**Keywords:** animal models, asthma, asthma treatment, pharmacology and pharmacogenomics, rhinitis

## DESCRIPTION OF INVENTION

Asthma is a chronic inflammatory disease characterized by recurrent airway obstruction, airway hyperresponsiveness (AHR), airway inflammation, and airway remodeling, which is often associated with allergy and allergic rhinitis. Many patients with asthma are poorly controlled by current drug treatment, particularly a subgroup of patients with difficult‐to‐treat severe asthma, characterized by chronic symptoms, severe exacerbations, progressive loss of lung function, and resistance to corticosteroids. New therapeutic options are therefore highly warranted.

Our patent covers arginase as a new drug target for the treatment of asthma and/or allergic rhinitis, making use of an arginase inhibitor.^1^ Arginase is the final enzyme of the hepatic urea cycle, converting l‐arginine to l‐ornithine and urea. Arginase is also expressed in nonhepatic tissues, including the airways. Two isoforms have been identified, arginases 1 and 2, which are encoded by different genes and are differentially expressed in the body.^2^

**Figure 1:**
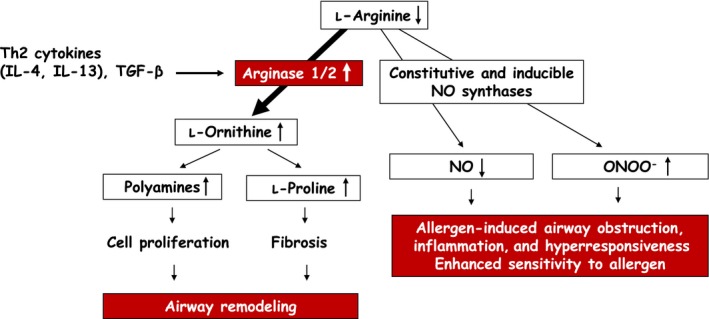
Pathways of l‐arginine metabolism and their relationship to allergen‐induced airway obstruction, airway inflammation, airway hyperresponsiveness and airway remodeling, and enhanced allergen sensitivity. Nitric oxide (NO) is synthesized from l‐arginine by constitutive and inducible NO synthases. NO has bronchodilatory and anti‐inflammatory actions and inhibits mediator release from mast cells. l‐Arginine is also metabolized to l‐ornithine and urea by arginases 1 and 2. Th2 cytokines (IL‐4 and IL‐13) and TGF‐β induce increased arginase expression and activity, which reduces the availability of l‐arginine to the NO synthases. This reduces the production of NO and induces production of superoxide anion (${\text{O}}_{2}^{-}$) by these enzymes, causing formation of their reaction product peroxynitrite (ONOO ^−^), which has pro‐contractile and pro‐inflammatory actions in the airways. Collectively, these processes contribute to allergen‐induced airway obstruction, airway inflammation and airway hyperresponsiveness, and increased sensitivity to the allergen. Furthermore, the increased synthesis of l‐ornithine from arginase provides a precursor for polyamines and l‐proline, which stimulate cell proliferation, and collagen production and fibrosis, respectively, causing airway remodeling. IL‐4, interleukin‐4; IL‐13, interleukin‐13; NO, nitric oxide; ONOO ^−^, peroxynitrite; TGF‐β, transforming growth factor‐β [Color figure can be viewed at wileyonlinelibrary.com]

Since l‐arginine is also substrate for constitutive and inducible nitric oxide synthases (cNOS and iNOS) yielding l‐citrulline and NO, one biological function of extrahepatic arginase may be regulating NO levels through competition with NOS for their common substrate^2^ (Figure 1). Under healthy conditions, NO, derived from cNOS in airway epithelium and inhibitory nonadrenergic‐noncholinergic (iNANC) nerves, has a protective role in the airways by inducing bronchodilation as well as inhibiting airway inflammation and mediator release from mast cells. In allergic asthma, arginases can be upregulated by Th2 cytokines (IL‐4, IL‐13) and TGF‐β, causing reduced cNOS‐derived NO production and increased production of pro‐contractile and pro‐inflammatory peroxynitrite (ONOO^−^) by particularly inflammation‐induced iNOS, by reduced bioavailability of l‐arginine to these enzymes. Moreover, increased arginase activity increases the production of l‐ornithine and its downstream products polyamines and l‐proline, which may be involved in airway remodeling by inducing cell proliferation, and enhanced collagen production and fibrosis, respectively^2^ (Figure 1).

Supporting evidence for this mechanism and thus for a role of arginase in the pathophysiology of allergic asthma and, potentially, other allergic disorders like allergic rhinitis was found in a guinea pig model of allergic asthma. Using this model, we discovered that inhalation of the potent specific arginase inhibitor 2(*S*)‐amino‐6‐boronohexanoic acid (ABH) considerably reduces the airway sensitivity to inhaled allergen and protects against allergen‐induced early and late asthmatic reactions, AHR after these reactions, and airway inflammation. Moreover, ABH acutely reversed AHR after the early and late asthmatic reaction.^3^ Based on the observed anti‐allergic, bronchoprotective, and anti‐inflammatory effects of ABH and indications that arginase may be involved in asthma and allergic rhinitis in patients, we claimed the use of an arginase inhibitor in the prophylactic maintenance treatment of patients with asthma and/or allergic rhinitis, by preventing the development of allergen‐induced upper and lower airway obstruction and AHR, wherein the arginase inhibitor is administered by topical inhalation.^1^

### Path leading to the invention and recent developments

In 1996, by performing perfusion experiments in intact airways from allergen‐challenged guinea pigs ex vivo, we demonstrated that a deficiency of cNOS‐derived NO may contribute to allergen‐induced AHR after the early asthmatic reaction.^4^ The mechanism of this NO deficiency was unknown. In 1997, we presented some of our data on NO deficiency and asthma on a symposium on the pharmacology of NO in Odense, Denmark. Coincidently, at the same meeting there was the first demonstration that inhibition of arginase by a *bona fide* arginase inhibitor increased NOS activity in rat alveolar macrophages.^5^ Although it took almost 10 years to obtain proof of concept,^2^, ^3^ it provided an important clue to the underlying mechanism of the allergen‐induced NO deficiency and the therapeutic potential of arginase inhibitors in asthma.

By using a novel potent and specific arginase inhibitor (N^ω^‐hydroxy‐nor‐l‐arginine), we demonstrated that arginase inhibition reduces guinea pig airway responsiveness in vitro by increasing NO production (see Ref. 2). In ex vivo studies, using a guinea pig model of allergic asthma, we discovered that arginase activity in the airways is increased after allergen challenge, causing AHR after the early asthmatic reaction by reducing the production of neuronal as well as non‐neuronal cNOS‐derived NO by reduced bioavailability of l‐arginine to the enzyme (Ref. 2). Moreover, we found evidence that AHR after the late asthmatic reaction is caused by arginase‐induced attenuation of l‐arginine availability to particularly iNOS, switching the enzyme to simultaneous production of NO and ${\text{O}}_{2}^{-}$ and, consequently, detrimental ONOO^−^ (Ref. 2). Collectively, these observations paved the way to the proof‐of‐concept in vivo study presented above.^3^ Whereas the bronchoprotective effect of ABH was anticipated based on the ex vivo studies, the anti‐allergic effect became apparent from the ~30‐fold higher allergen dose needed to induce airway obstruction. More recently, we confirmed a role for arginase in airway remodeling by demonstrating that arginase inhibition attenuated airway smooth muscle hyperplasia, airway fibrosis, mucosal gland hypertrophy, and goblet cell hyperplasia following repeated allergen exposure^6^ (Figure 1).

There is growing evidence for an important role of arginase in patients with asthma. Arginase 1 and arginase 2 expression and/or arginase activity are enhanced in asthmatic airways and in serum, and there is an association between arginase expression in bronchial brushings, serum arginase activity, plasma l‐arginine, and metabolite concentration and disease severity (lung function and Fe(NO); Refs. 2 and 7). Moreover, *ARG1* and *ARG2* polymorphisms are associated with asthma, asthma severity (lung function, AHR), and reduced responsiveness to β_2_‐agonists and glucocorticosteroids.^8^ In addition, enhanced expression of arginases 1 and 2 in nasal mucosa and increased arginase activity in serum have recently been found in patients with allergic rhinitis (Ref. 7).

## CONCLUSION

Studies in animal models and in asthmatic patients indicate an important role for both arginase 1 and arginase 2 in the pathophysiology of, particularly severe, asthma and allergic rhinitis. Therefore, arginase inhibitors, having an unique anti‐allergic, bronchoprotective, anti‐inflammatory, and anti‐remodeling profile, may be effective in the treatment of these diseases, possibly guided by the arginine metabolome in blood. Potential drugs are presently under development, some in clinical trials. As no subtype‐selective arginase inhibitors are presently available, development of such selective inhibitors to address potential, as yet unknown, differential roles of arginases 1 and 2 may further benefit patients suffering from these diseases.

## CONFLICTS OF INTEREST

Dr. Meurs reports grants from Lung Foundation Netherlands, grants from N.V. Organon, Oss, The Netherlands, grants from Schering‐Plough Research Institute, Oss, The Netherlands, grants from Merck Sharpe and Dohme, Oss, The Netherlands, during the conduct of the study; grants from Netherlands Organisation for Scientific Research (NWO), grants from Carmolex Inc., Pittsburgh, PA, USA, outside the submitted work; In addition, Dr. Meurs has a patent Use of arginase inhibitors in the treatment of asthma and allergic rhinitis licensed to Carmolex Inc., Pittsburgh, PA, USA. Dr. Zaagsma reports grants from Lung Foundation Netherlands, grants from N.V. Organon, Oss, The Netherlands, grants from Schering‐Plough Research Institute, Oss, The Netherlands, grants from Merck Sharpe and Dohme, Oss, The Netherlands, during the conduct of the study; In addition, Dr. Zaagsma has a patent Use of arginase inhibitors in the treatment of asthma and allergic rhinitis licensed to Carmolex Inc., Pittsburgh, PA, USA. Dr. Maarsingh reports grants from N.V. Organon, Oss, The Netherlands, grants from Schering‐Plough Research Institute, Oss, The Netherlands, personal fees from Schering‐Plough, Kenilworth, NJ, USA, grants from Merck Sharpe and Dohme, Oss, The Netherlands, during the conduct of the study; In addition, Dr. Maarsingh has a patent Use of arginase inhibitors in the treatment of asthma and allergic rhinitis licensed to Carmolex Inc., Pittsburgh, PA, USA. At the time of this work Dr. van Duin was an employee of Organon/Schering Plough/Merck MSD and involved in the scientific collaboration with the corresponding author, Dr. Meurs. In addition, Dr. van Duin has a patent Use of arginase inhibitors in the treatment of asthma and allergic rhinitis licensed to Carmolex Inc., Pittsburgh, PA, USA.
